# Editorial: Importance of quality assurance in radiation oncology clinical trials

**DOI:** 10.3389/fonc.2026.1841054

**Published:** 2026-06-11

**Authors:** TJ FitzGerald, Fran Laurie, Matthew Iandoli, Koren Smith, Maryann Bishop-Jodoin

**Affiliations:** 1 Department of Radiation Oncology, University of Massachusetts Chan Medical School, Worcester, MA, United States; 2 Imaging and Radiation Oncology Core (IROC) Rhode Island/Quality Assurance Review Center (QARC), Department of Radiation Oncology, University of Massachusetts Chan Medical School, Lincoln, RI, United States

**Keywords:** clinical trials, oncology, patient care, process improvement, radiation therapy

For process improvements to occur in oncology clinical medicine, well-constructed clinical trials have become a standard component in patient care. To demonstrate improvements in tumor control and assessment of the impact of therapy on normal tissue function, clinical trials have become the most respected vehicle for the evolution of adjustments and improvements in clinical management. There are challenges to developing and executing clinical trials. To be successful, trials must ask relevant questions and be constructed in a manner that the trial questions can be managed and answered in a cooperative multi-institutional/site setting. Most trials require multiple diverse institutions to actively participate in the trial to meet accrual goals for statistical relevance. To further add to the complexity of trial management, many clinical trials today both in industry and the cooperative groups accrue patients with worldwide participation. Institutions have various standards of management, and often site investigators need to adjust how they may treat their patients not enrolled on a cancer clinical trial. The process of establishing guidelines for patient management in a clinical trial and the management of maintaining symmetry and uniformity of patient care management in the clinical trial for each patient entered is the goal of clinical trial quality assurance (QA).

An oncology clinical trials QA program has five major components: site qualification, site credentialing, data acquisition, data management, and radiation therapy (RT) and diagnostic imaging QA review, with each component playing an important role in successful trial management. Individual therapy sites and investigators need to demonstrate they possess all necessary infrastructure and personnel to qualify for participation in clinical trials. This may include a portfolio of personnel and equipment. For example, gynecologic oncology trials will require an institution to have a complete portfolio of brachytherapy services to participate as well as a full array of teletherapy services. The QA program needs to provide a robust infrastructure for clinical trial design support. In addition to writing guidelines for trial objectives, support for trial and site investigators is ongoing during and after completion of the trial. Often questions and issues arise requiring the adjustment of guidelines.

Credentialing for individual clinical trials is a step beyond qualification. Specific trials may require validation of specific site technology. This can include a phantom credentialing process or a knowledge test to demonstrate and confirm image acquisition process, ability to contour targets, plan development/execution, and electronic data transfer. These elements will play an essential role in the development of clinical trial QA processes for radiopharmaceutical trials as, unlike trials using teletherapy, radiopharmaceutical treatment delivery cannot be reviewed in real time. Therefore, credentialing will include data management tools and computational platforms to ensure the correct data sets are transferred, and computational metrics for dose volume analysis are uniform. There are times when credentialing can overlap into multiple trials. For example, once a site investigative team has demonstrated proficiency in intensity modulation therapy, they are not required to repeat that proficiency for other trials using intensity modulation. If a trial requires a specific skill in contouring an object, this can be incorporated as a credentialing vehicle into a specific trial despite approval for intensity modulation. For example, trials in central nervous system RT have included the hippocampus as a conformal avoidance structure. Intensity modulation is required to successfully create a plan for conformal avoidance of the hippocampus however a separate credentialing module was developed to demonstrate proficiency in contouring the hippocampus as an avoidance object. Therefore, credentialing can be specific for a clinical trial, however QA centers work to minimize duplication of effort to ensure feasibility of trial accrual and limit burden on site investigators.

Data acquisition and data management are evolving into essential components of trials. As clinical trials become more complex with response adaptive components including multiple points of randomization within a single trial, acquiring the correct information at the correct time point is important to ensure data can be reviewed in real time to respond to the specific trial objectives. For example, case review for modern Wilms tumor studies is important. In stage 4 patients with pulmonary involvement at presentation, thoracic RT is not delivered if the patient has a complete response to chemotherapy. The cases are reviewed by a limited number of trial radiologists in real time providing uniformity in interpretation. However, the data are received continuously, often in diverse formats which must be processed and collated in a uniform manner for this review. The work is ongoing and dynamic with interactions between site and trial investigators. These processes continue to mature with increased complexity of modern clinical trials.

Clinical trials require the acquisition of a significant amount of data including medical history, laboratory data, pathology, biomarkers, imaging, RT treatment objects, systemic therapy, and molecular biology. These components play an important role in patient outcome and outcome analysis. One essential objective of QA is to affirm the data is accurate and managed in a compliant manner. These data should be managed so that the information can be re-purposed for additional study and review by independent investigators and audit teams. Because patients are treated in a uniform format and the objects are acquired in a comprehensive trial- compliant manner, the data becomes an ideal resource for the development of platforms for artificial intelligence and secondary research for questions not anticipated at the time of trial design.

The work touches on all topics presented in this research portfolio. Accurate and complete datasets provide confidence we can trust the outcome of an oncology clinical trial (1–6).

## References

[1] FitzGerald TJ Urie M Ulin K Laurie F Yorty J Hanusik R . Processes for quality improvements in radiation oncology clinical trials. Int J Radiat Oncol Biol Phys. (2008) 71:S76–9. doi: 10.1016/j.ijrobp.2007.07.2387 18406943 PMC2391004

[2] FitzGerald TJ Bishop-Jodoin M Followill DS Galvin J Knopp MV Michalski JM . Imaging and data acquisition in clinical trials for radiation therapy. Int J Radiat Oncol Biol Phys. (2016) 94:404–11. doi: 10.1016/j.ijrobp.2015.10.028 26853346 PMC4746496

[3] Fitzgerald TJ Bishop-Jodoin M Bosch WR Curran WJ Followill DS Galvin JM . Future vision for the quality assurance of oncology clinical trials. Front Oncol. (2013) 3:31. doi: 10.3389/fonc.2013.00031 23508883 PMC3598226

[4] FitzGerald TJ Followill D Laurie F Boterberg T Hanusik R Kessel S . Quality assurance in radiation oncology. Pediatr Blood Cancer. (2021) 68 Suppl 2:e28609. doi: 10.1002/pbc.28609 33818891 PMC10578132

[5] FitzGerald TJ . A new model for imaging and radiation therapy quality assurance in the National Clinical Trials Network of the National Cancer Institute. Int J Radiat Oncol Biol Phys. (2014) 88:272–3. doi: 10.1016/j.ijrobp.2013.09.030 24411600

[6] NIH: National Cancer Institute . NCTN: NCI’s National Clinical Trials Network (2026). Available online at: https://www.cancer.gov/research/areas/clinical-trials/nctn (Accessed March 22, 2026).

